# Bovine Lactoferrin Modulates Mononuclear Cell Activity in Human Palatine Tonsils

**DOI:** 10.3390/ijms27052442

**Published:** 2026-03-06

**Authors:** Takumi Yago, Chisane Kujirai, Hirotsugu Oda, Takahiro Inoue, Hisataka Ominato, Risa Wakisaka, Ryosuke Sato, Michihisa Kono, Hidekiyo Yamaki, Kenzo Ohara, Takumi Kumai, Miyuki Tanaka, Miki Takahara

**Affiliations:** 1 R&D Division, Morinaga Milk Industry Co., Ltd., 5-1-83 Higashihara, Zama 252-8583, Kanagawa, Japan; 2 Department of Otolaryngology-Head and Neck Surgery, Asahikawa Medical University, 1-1-1 Midorigaoka Higashi 2-jo, Asahikawa 078-8510, Hokkaido, Japanh-ominato@asahikawa-med.ac.jp (H.O.); rsato@asahikawa-med.ac.jp (R.S.); miki@asahikawa-med.ac.jp (M.T.)

**Keywords:** bovine lactoferrin, human palatine tonsils, tonsillar mononuclear cells, immune cells, plasmacytoid dendritic cells, interferon, Toll-like receptor, recurrent tonsillitis, immunoglobulin A nephropathy

## Abstract

Lactoferrin (LF) is present in tears, nasal secretions, saliva, and milk and maintains mucosal homeostasis. The palatine tonsils represent the first immune tissue to recognize pathogens invading the oral cavity via Toll-like receptors (TLRs). We aimed to investigate the effects of bovine LF on tonsillar immune cells stimulated with ligands of TLR7 or TLR9, which recognize viral single-stranded RNA or bacterial unmethylated CpG DNA. Mononuclear cells isolated from palatine tonsils of patients with recurrent tonsillitis or immunoglobulin A (IgA) nephropathy were cultured with LF, TLR7, or TLR9 ligands. Under TLR7 stimulation, LF enhanced the activation of plasmacytoid dendritic cells (pDCs), T-killer cells, and B cells without inducing inflammatory cytokines. In contrast, under TLR9 stimulation, LF suppressed the activation of pDCs, myeloid dendritic cells, T-helper cells, T-killer cells, B cells, and natural killer cells, as well as the production of TNF-α and IL-6. Moreover, LF decreased the production of the B-cell activation factor (BAFF), a proliferation-inducing ligand (APRIL), and galactose-deficient IgA1, all of which are risk factors of IgA nephropathy. Overall, LF may enhance the immune response against viruses and contribute to immune tolerance against commensal bacteria in the palatine tonsils, indicating potential benefits in managing cold-like symptoms, recurrent tonsillitis, and IgA nephropathy.

## 1. Introduction

The palatine tonsils constitute a part of the nasal-associated lymphoid tissue (NALT) forming Waldeyer’s ring and serve as a first-line immune defense against pathogens entering the oral cavity [1]. Immune cells residing in the palatine tonsils express various Toll-like receptors (TLRs), which recognize microbial components and initiate innate and adaptive immune responses [2]. Among these immune cells, plasmacytoid dendritic cells (pDCs) isolated from palatine tonsils respond to ligands of TLR7, which recognizes viral single-stranded RNA (ssRNA), and TLR9, which recognizes bacterial unmethylated deoxycytidyl-deoxyguanosine oligodeoxynucleotides (CpG-ODNs), leading to cellular activation and cytokine production [3]. However, excessive or chronic stimulation of TLRs by bacterial or viral components can disrupt immune homeostasis and increase the risk of inflammatory and autoimmune diseases [4].

Recurrent tonsillitis (RT) is characterized by repeated inflammation of the palatine tonsils caused by persistent bacterial or viral infections, with *Streptococcus pyogenes* being the predominant causative pathogen [5]. In immunoglobulin A (IgA) nephropathy (IgAN), excessive production of aberrantly glycosylated IgA in the tonsils results in its deposition within the renal glomeruli, leading to inflammation and tissue damage. This pathological process is associated with excessive immune responses to bacterial unmethylated CpG-ODN within the tonsils [6]. Surgical removal of the tonsils (tonsillectomy) is an established therapeutic option for both RT and IgAN; however, this procedure requires general anesthesia and is invasive, underscoring the need for less invasive strategies to modulate tonsillar immune responses [7,8].

Lymphocytes activated in different mucosa-associated lymphoid tissues (MALTs) exhibit distinct homing properties. Lymphocytes induced in gut-associated lymphoid tissues (GALTs), such as Peyer’s patches, primarily migrate to the mucosa of the small and large intestines, whereas lymphocytes induced in NALTs, including the palatine tonsils, mainly home to effector sites in the upper respiratory tract, lacrimal and salivary glands, and the systemic immune system. Therefore, immunomodulation at the level of the palatine tonsils may exert not only local effects but also broader impacts on upper respiratory and systemic immunity [9].

Lactoferrin (LF) is an iron-binding glycoprotein present in milk and various exocrine secretions, including tears, nasal secretions, saliva, and vaginal secretions. Mucosal surfaces covered by these secretions are continuously exposed to environmental pathogens, suggesting that LF plays a crucial role in the host defense mechanism against invading pathogens on the mucosal surfaces of the eye, nasal cavity, oral cavity, and vagina. Bovine LF, derived from cow’s milk, is widely used as a functional food ingredient [10]. Oral ingestion of LF alleviates cold-like symptoms [11] and reduces the incidence of acute gastrointestinal symptoms in healthy adults [12].

One of the mechanisms by which LF contributes to the host defense against invading pathogens is the modulation of TLR-mediated signaling pathways. LF enhances the recognition of TLR7 ligands within endosomes and subsequently augments interferon (IFN) signaling in human peripheral blood mononuclear cell (PBMC)-derived pDCs [13]. In contrast, LF inhibits TLR9-mediated IFN-α production in pDCs induced by bacterial CpG-ODN [14] and suppresses inflammatory responses in monocytes triggered by Epstein–Barr virus (EBV) via TLR9 signaling [15]. These findings indicate that LF can differentially regulate immune responses by interacting with TLRs or their ligands.

Although the immunomodulatory effects of LF have been extensively investigated in immune cells derived from peripheral blood [13,16] and GALT, such as Peyer’s patches in the small intestine [17,18], its effects on immune cells residing in NALTs remain unclear. Given that the palatine tonsils are among the first NALTs to encounter orally ingested LF, elucidating its immunological impact at this site is of particular importance.

In the present study, we aimed to clarify the effects of LF on immune cells in the palatine tonsils. Specifically, we investigated the effects of LF on mononuclear cells isolated from the palatine tonsils derived from patients with RT or IgAN stimulated with TLR7 ligands to mimic ssRNA viral stimulation and with TLR9 ligands to mimic bacterial stimulation.

## 2. Results

### 2.1. Inflammatory Cytokine Production from Tonsillar Mononuclear Cells (TMCs) Under TLR7 and TLR9 Stimulation Following LF Treatment

**Figure 1:**
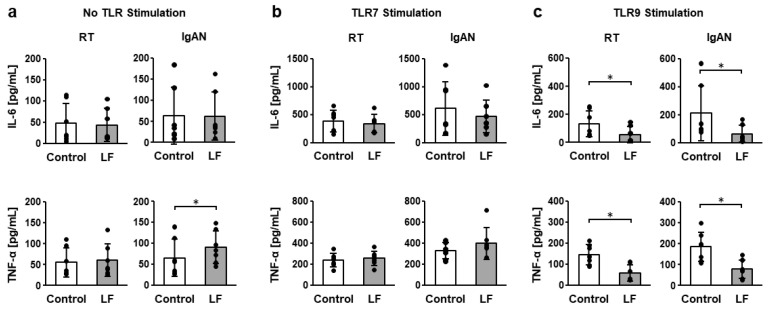
Inflammatory cytokine concentration in the culture supernatants of tonsillar mononuclear cells (TMCs). (**a**) Interleukin (IL)-6 and tumor necrosis factor (TNF)-α concentrations under no TLR stimulation. (**b**) IL-6 and TNF-α concentrations under TLR7 stimulation. (**c**) IL-6 and TNF-α concentrations under TLR9 stimulation. After overnight incubation of TMCs from recurrent tonsillitis (RT) and immunoglobulin A nephropathy (IgAN) patients with 100 µg/mL lactoferrin (LF) in the presence or absence of 10 µg/mL R-848 or 1 µM deoxycytidyl-deoxyguanosine oligonucleotides (CpG-ODN), IL-6, and TNF-α concentration in culture supernatants were measured using enzyme-linked immunosorbent assays (ELISAs). White bars represent the control group, and gray bars represent the LF-treated group. Values are presented as the mean and SD. Black circles represent individual values. *n* = 7. * Significantly different from the control group (*p* < 0.05, paired Student’s *t*-test).

Figure 1 and Figure S1 show interleukin (IL)-6 and tumor necrosis factor (TNF)-α concentrations in the culture supernatants of TMCs from patients with recurrent tonsillitis (RT) and IgA nephropathy (IgAN) following incubation with LF, R-848 (a TLR7 agonist), and CpG-ODN (a TLR9 agonist). The addition of R-848 or CpG-ODN significantly increased IL-6 and TNF-α concentrations in TMCs derived from both RT and IgAN (Figure S1a,b). Under no stimulation, LF treatment significantly increased the TNF-α concentration in IgAN TMCs (Figure 1a). Under TLR7 stimulation, LF treatment exhibited no effect on IL-6 and TNF-α concentrations in either RT or IgAN TMCs (Figure 1b). However, under TLR9 stimulation, LF treatment significantly suppressed the IL-6 and TNF-α concentrations in both RT and IgAN TMCs (Figure 1c).

### 2.2. The Cell Composition of TMCs from Patients with RT and IgAN

The frequencies of pDCs, myeloid dendritic cells (mDCs), T-helper cells, T-killer cells, B cells, and natural killer (NK) cells in TMCs from patients with RT and IgAN are shown in Figure S2. The proportions of pDCs, T-helper cells, T-killer cells, and NK cells in TMCs were significantly higher in patients with IgAN than in those with RT (Figure S2a,c,d,f). In contrast, the proportion of B cells was significantly lower in patients with IgAN (Figure S2e).

### 2.3. Immune-Cell Activity Under TLR7 and TLR9 Stimulation

The effects of TLR7 and TLR9 stimulation on activation-marker expression in pDCs, mDCs, T cells, B cells, and NK cells in TMCs from patients with RT and IgAN are shown in Figure S3. The addition of R-848 or CpG-ODN resulted in higher expression levels of activation markers across the immune-cell subsets. In particular, significant increases in expression levels were observed—in pDCs for cell-surface HLA-DR, cell-surface CD86, intracellular IFN-α, intracellular B-cell activation factor (BAFF), and intracellular a proliferation-inducing ligand (APRIL) in both RT and IgAN TMCs (Figure S3a); in mDCs for cell-surface HLA-DR in IgAN TMCs and cell-surface CD86 in RT and IgAN TMCs (Figure S3b); in T-helper cells for cell-surface CD69 in RT and IgAN TMCs (Figure S3c); in T-killer cells for cell-surface CD69 in RT and IgAN TMCs (Figure S3d); in B cells for cell-surface CD69 and CD86 and intracellular IgA in RT and IgAN TMCs (Figure S3e); and in NK cells for cell-surface CD69 and intracellular IFN-γ in IgAN TMCs (Figure S3f).

### 2.4. The Cellular Uptake of LF in TMCs

The fluorescein isothiocyanate (FITC) fluorescence signals of pDCs, mDCs, T-helper cells, T-killer cells, B cells, and NK cells in TMCs are shown in Figures S4 and S5. The addition of FITC-labeled LF led to increased FITC fluorescence signals in all immune-cell subsets (Figure S4), whereas pretreatment with a nucleolin-neutralizing antibody attenuated these signals (Figure S5).

### 2.5. Immune-Cell Activity Following LF Treatment Without TLR Stimulation

**Figure 2:**
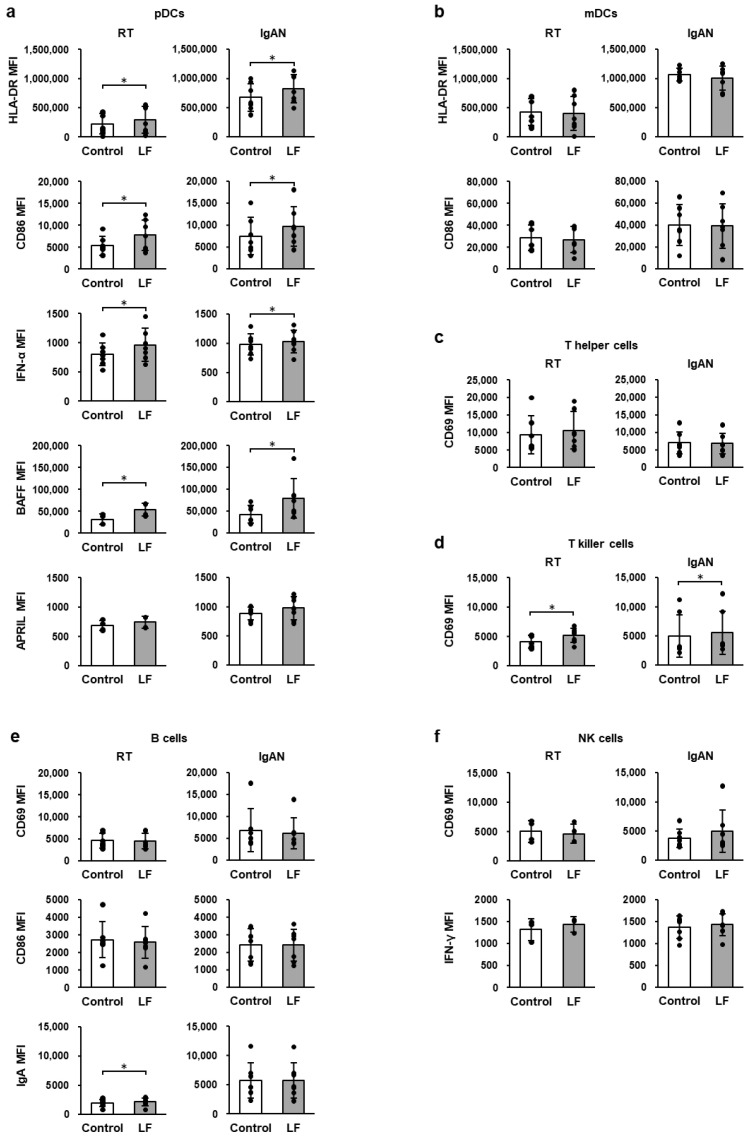
Expression levels of activation markers in the immune cells. (**a**) Cell-surface human leukocyte antigen (HLA)-DR, CD86, intracellular IFN-α, B-cell activation factor (BAFF), and a proliferation-inducing ligand (APRIL) in plasmacytoid dendritic cells (pDCs, CD123+CD304+ cells). (**b**) Cell-surface HLA-DR and CD86 in myeloid dendritic cells (mDCs, CD1c+CD11c+ cells). (**c**) Cell-surface CD69 in T-helper cells (CD3+CD4+ cells). (**d**) Cell-surface CD69 in T-killer cells (CD3+CD8+ cells). (**e**) Cell-surface CD69, CD86, and intracellular immunoglobulin A (IgA) in B cells (CD19+ cells). (**f**) Cell-surface CD69 and intracellular IFN-γ in natural killer (NK) cells (CD3−CD56+ cells). After overnight incubation of TMCs from RT and IgAN patients with 100 µg/mL LF, the expression levels of immune-cell activity markers were measured using flow cytometry. White bars represent the control group, and gray bars represent the LF-treated group. Values are presented as the mean and SD. Black circles represent individual values. *n* = 3–7. * Significantly different from the control group (*p* < 0.05, paired Student’s *t*-test). IgAN, immunoglobulin A nephropathy; LF, lactoferrin; MFI, geometric mean fluorescence intensity; RT, recurrent tonsillitis; TMCs, tonsillar mononuclear cells.

Figure 2 shows the expression of the activation markers in TMCs from patients with RT and IgAN without TLR stimulation. In the absence of TLR ligands, LF treatment significantly increased the expression levels of cell-surface HLA-DR, CD86, intracellular IFN-α, and BAFF in pDCs (Figure 2a); cell-surface CD69 in T-killer cells in TMCs from RT and IgAN patients (Figure 2d); and intracellular IgA in B cells in RT TMCs (Figure 2e). In contrast, LF treatment did not affect the expression of activation markers in the mDCs, T-helper cells, or NK cells (Figure 2b,c,f).

### 2.6. Immune-Cell Activity Following LF Treatment Under TLR7 Stimulation

**Figure 3:**
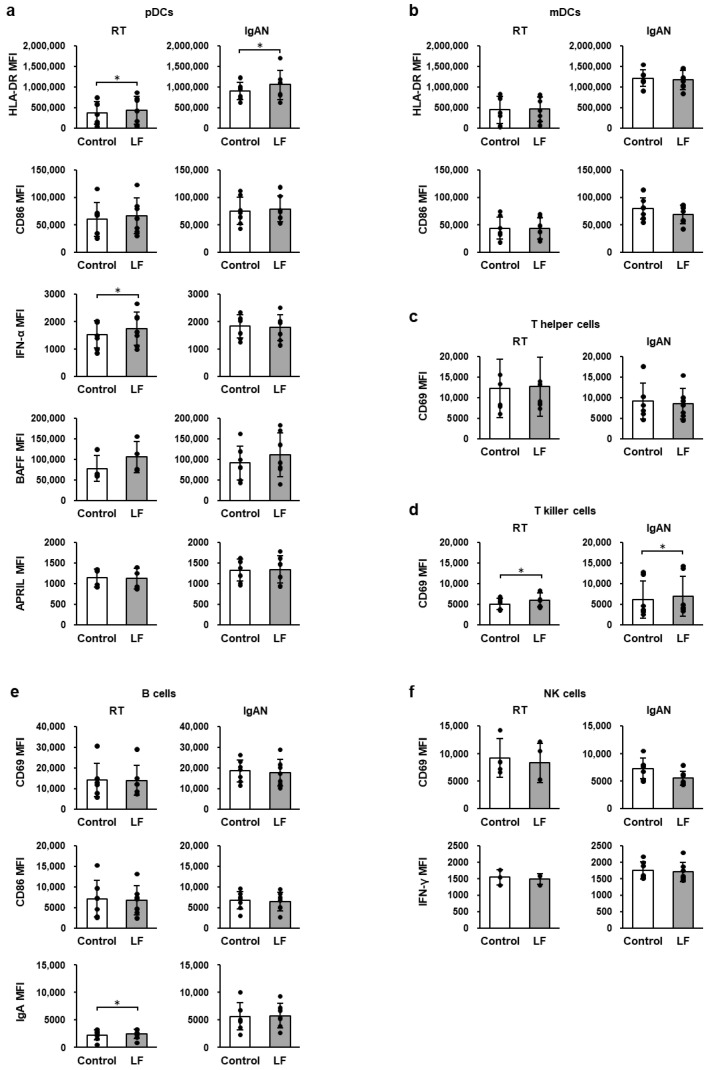
Expression levels of activation markers in the immune cells under TLR7 stimulation. (**a**) Cell-surface HLA-DR, CD86, intracellular IFN-α, BAFF, and APRIL in pDCs. (**b**) Cell-surface HLA-DR and CD86 in mDCs. (**c**) Cell-surface CD69 in T-helper cells. (**d**) Cell-surface CD69 in T-killer cells. (**e**) Cell-surface CD69, CD86 and intracellular IgA in B cells. (**f**) Cell-surface CD69 and intracellular IFN-γ in NK cells. After overnight incubation of TMCs from RT and IgAN patients with 100 µg/mL LF and 10 µg/mL R-848, the expression levels of immune-cell activity markers were measured using flow cytometry. White bars represent the control group, and gray bars represent the LF-treated group. Values are presented as the mean and SD. Black circles represent individual values. *n* = 3–7. * Significantly different from the control group (*p* < 0.05, paired Student’s *t*-test). IgAN, immunoglobulin A nephropathy; mDC, myeloid dendritic cell; LF, lactoferrin; MFI, geometric mean fluorescence intensity; NK, natural killer; pDC, plasmacytoid dendritic cell; RT, recurrent tonsillitis; TMCs, tonsillar mononuclear cells.

Figure 3 shows the expression of activation markers in TMCs from patients with RT and IgAN under TLR7 stimulation. In the presence of R-848, LF treatment significantly enhanced the expression levels of cell-surface HLA-DR in pDCs and cell-surface CD69 in T-killer cells in TMCs from RT and IgAN patients, as well as intracellular IFN-α in pDCs and intracellular IgA in B cells in RT TMCs (Figure 3a,d,e). In contrast, LF treatment did not affect the expression of activation markers in mDCs, T-helper cells, or NK cells (Figure 3b,c,f).

### 2.7. Immune-Cell Activity Following LF Treatment Under TLR9 Stimulation

**Figure 4:**
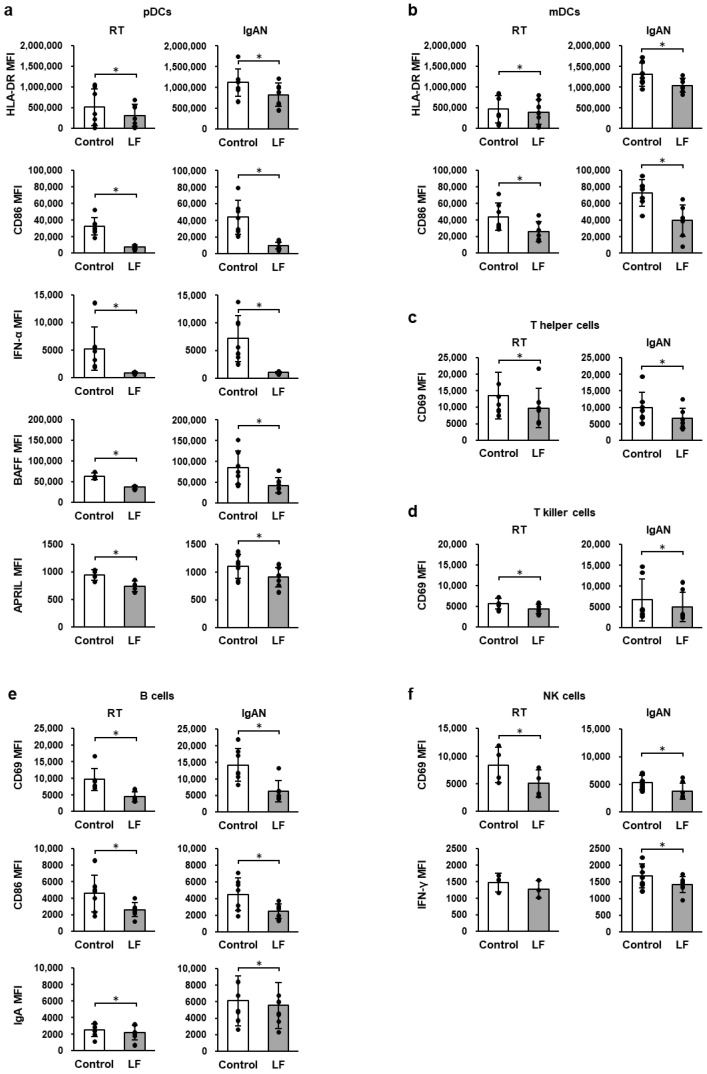
Expression levels of activation markers in the immune cells under TLR9 stimulation. (**a**) Cell-surface HLA-DR, CD86, intracellular IFN-α, BAFF, and APRIL in pDCs. (**b**) Cell-surface HLA-DR and CD86 in mDCs. (**c**) Cell-surface CD69 in T-helper cells. (**d**) Cell-surface CD69 in T-killer cells. (**e**) Cell-surface CD69, CD86 and intracellular IgA in B cells. (**f**) Cell-surface CD69 and intracellular IFN-γ in NK cells. After overnight incubation of TMCs from RT and IgAN patients with 100 µg/mL LF and 1 µM CpG-ODN, the expression levels of immune-cell activity markers were measured using flow cytometry. White bars represent the control group, and gray bars represent the LF-treated group. Values are presented as the mean and SD. Black circles represent individual values. *n* = 3–7. * Significantly different from the control group (*p* < 0.05, paired Student’s *t*-test). CpG-ODN, deoxycytidyl-deoxyguanosine oligodeoxynucleotide; IgAN, immunoglobulin A nephropathy; LF, lactoferrin; mDC, myeloid dendritic cell; MFI, geometric mean fluorescence intensity; NK, natural killer; pDC, plasmacytoid dendritic cell; RT, recurrent tonsillitis; TMCs, tonsillar mononuclear cells.

Figure 4 shows the expression of activation markers in TMCs from patients with RT and IgAN under TLR9 stimulation. In the presence of CpG-ODN, LF treatment significantly suppressed the expression levels of cell-surface HLA-DR, CD86, intracellular IFN-α, BAFF, and APRIL in pDCs (Figure 4a); cell-surface HLA-DR and CD86 in mDCs (Figure 4b); cell-surface CD69 in T-killer cells and T-helper cells (Figure 4c,d); cell-surface CD69, CD86, and intracellular Ig in B cells (Figure 4e); and cell-surface CD69 in NK cells (Figure 4f) in TMCs from patients with RT and IgAN. Additionally, it significantly suppressed intracellular IFN-γ expression in NK cells in IgAN TMCs (Figure 4f).

### 2.8. IgAN-Associated Pathogenic Factors Production from TMCs Under TLR7 and TLR9 Stimulation Following LF Treatment

**Figure 5:**
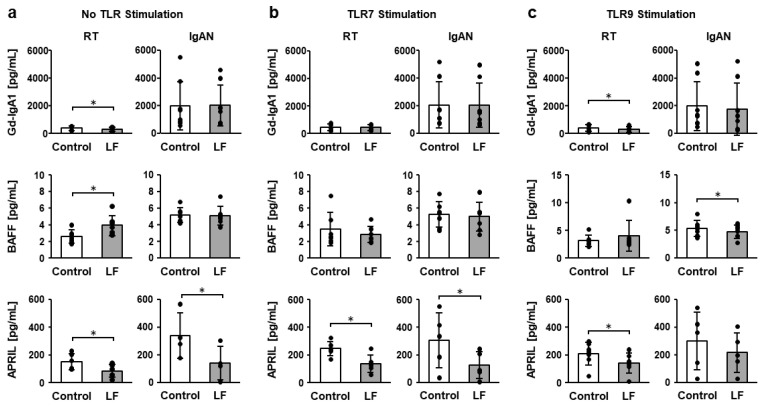
Galactose-deficient IgA1 (Gd-IgA1), BAFF, and APRIL concentrations in the culture supernatants of TMCs. (**a**) Gd-IgA1, BAFF, and APRIL concentrations under no TLR stimulation. (**b**) Gd-IgA1, BAFF, and APRIL concentrations under TLR7 stimulation. (**c**) Gd-IgA1, BAFF, and APRIL concentrations under TLR9 stimulation. After overnight incubation of TMCs from RT and IgAN patients with 100 µg/mL lactoferrin (LF) in the presence or absence of 10 µg/mL R-848 or 1 µM CpG-ODN, the concentrations of Gd-IgA1, BAFF, and APRIL in culture supernatants were measured using ELISA. White bars represent the control group, and gray bars represent the LF-treated group. Values are presented as the mean and SD. Black circles represent individual values. *n* = 3–8. * Significantly different from the control group (*p* < 0.05, paired Student’s *t*-test). CpG-ODN, deoxycytidyl-deoxyguanosine oligodeoxynucleotide; IgAN, immunoglobulin A nephropathy; LF, lactoferrin; RT, recurrent tonsillitis; TMCs, tonsillar mononuclear cells.

Figure 5 shows the galactose-deficient IgA1 (Gd-IgA1), BAFF, and APRIL concentrations in the culture supernatants of RT and IgAN TMCs following incubation with LF, R-848, and CpG-ODN. In the absence of TLR stimulation, LF treatment significantly increased the BAFF concentration in RT TMCs and reduced Gd-IgA1 in RT TMCs and APRIL in RT and IgAN TMCs (Figure 5a). Under TLR7 stimulation, LF treatment significantly suppressed the APRIL concentration in RT and IgAN TMCs (Figure 5b). Under TLR9 stimulation, LF treatment significantly suppressed Gd-IgA1 and APRIL concentrations in RT TMCs and showed a tendency to suppress their concentrations (*p* < 0.1) in IgAN TMCs (Figure 5c). Additionally, LF treatment suppressed BAFF concentrations in IgAN TMCs (Figure 5c). 

## 3. Discussion

We investigated the effects of bovine LF on immune cells in palatine tonsils stimulated with ligands of TLR7, which recognizes viral ssRNA, or TLR9, which recognizes bacterial unmethylated CpG-DNA. In the tonsillar mononuclear cells (TMCs) of patients with recurrent tonsillitis (RT), TLR7 and TLR9 stimulation increased the expression of HLA-DR, CD86, IFN-α, BAFF and APRIL in pDCs; CD86 in mDCs; CD69 in T-helper and T-killer cells; and CD69, CD86 and IgA in B cells. In the TMCs of patients with IgA nephropathy (IgAN), TLR7 and TLR9 stimulation increased the expression of HLA-DR, CD86, IFN-α, BAFF, and APRIL in pDCs; HLA-DR and CD86 in mDCs; CD69 in T-helper and T-killer cells; CD69, CD86, and IgA in B cells; and CD69 and IFN-γ in NK cells. These findings suggest that viral and bacterial stimuli simultaneously activate various immune cells in complex cellular networks of TMCs.

TMCs contain higher proportions of pDCs and B cells and lower proportions of mDCs and T cells than PBMCs [3]. Additionally, NK cells constitute only a minor population, accounting for approximately 0.4% of the mononuclear cells isolated from tonsils [19]. IFN-α enhances BAFF and APRIL production in pDCs, and these factors induce IgA production by B cells [20]. IFN-α upregulates HLA-DR and CD86 expression in mDCs [21]; CD69 expression in T-killer cells and T-helper cells [22]; CD69 and IFN-γ expression in NK cells [23]; and CD69 and CD86 expression in B cells [24]. Therefore, IFN-α derived from pDCs may affect the activity of immune cells in TMCs.

R-848, a TLR7 ligand, selectively activates TLR7 and TLR8 in humans [25]. pDCs and B cells express TLR7, and mDCs express TLR8; therefore, these immune cells in TMCs may be activated independently or interactively by R-848. Additionally, pDCs and B cells express TLR9, and class A CpG-ODN (a TLR9 ligand) strongly induces IFN-α production in pDCs; however, they are poor stimulators of human B cells [26]. Therefore, the activation of immune cells in the TMCs by class A CpG-ODN is likely attributable to IFN-α produced by pDCs. FITC-labeled LF appeared to be incorporated into immune cells in TMCs via a LF receptor, suggesting that LF may directly act on these cells.

In the absence of TLR stimulation, LF enhanced the expression of HLA-DR, CD86, IFN-α, and BAFF in pDCs; CD69 in T-killer cells; and IgA in B cells of RT TMCs. Additionally, LF decreased Gd-IgA1 and APRIL and increased BAFF in the culture supernatant. Under TLR7 stimulation, LF enhanced the expression of HLA-DR and IFN-α in pDCs, CD69 in T-killer cells, and IgA in B cells. Additionally, LF decreased APRIL levels in the culture supernatant. Under TLR9 stimulation, LF inhibited the expression of HLA-DR, CD86, IFN-α, BAFF, and APRIL in pDCs; HLA-DR and CD86 in mDCs; CD69 in T-helper and T-killer cells; CD69, CD86, and IgA in B cells; and CD69 in NK cells. Additionally, LF decreased Gd-IgA1, APRIL, IL-6, and TNF-α in the culture supernatant.

In the absence of TLR stimulation, LF enhanced the expression of HLA-DR, CD86, IFN-α, and BAFF in pDCs and CD69 in T killer cells of IgAN TMCs. Additionally, LF decreased APRIL and increased TNF-α levels in the culture supernatant. Upon TLR7 stimulation, LF enhanced the expression of HLA-DR in pDCs and CD69 in T-killer cells. Additionally, LF decreased APRIL levels in the culture supernatant. Under TLR9 stimulation, LF inhibited the expression of HLA-DR, CD86, IFN-α, BAFF, and APRIL in pDCs; HLA-DR and CD86 in mDCs; CD69 in T-helper and T-killer cells; CD69, CD86, and IgA in B cells; and CD69 and IFN-γ in NK cells. Additionally, LF decreased BAFF, IL-6 and TNF-α and tended to decrease Gd-IgA1 and APRIL in the culture supernatant.

In our earlier study, LF enhanced IFN-α production in pDCs in PBMCs under TLR7 stimulation but not in the absence of TLR stimulation [16]. However, LF enhanced IFN-α production in pDCs in TMCs in this study, even in the absence of TLR stimulation. A possible reason for this is the different characteristics of immune cells in the peripheral blood and digestive-tract mucosa. Another reason is that TMCs are intrinsically activated with environmental viruses; viruses have been detected in excised palatine tonsils [27]. As viral ssRNA is negatively charged and LF is positively charged, they may form complexes and stimulate TLR7 more strongly than ssRNA alone. This enhancement of IFN production was also observed in TLR3 stimulated with LF and double-stranded RNA, an intermediate of viral replication [28] possibly explaining the mechanisms of the antiviral immune response of LF.

In contrast, LF binds to TLR9 coreceptor CD14 and inhibits the ability of TLR9 to recognize double-stranded DNA, thereby suppressing EBV-induced inflammation [15]. Moreover, LF binds to TLR9 agonist CpG-ODN and inhibits its stimulatory effect [29]. These findings suggest that LF suppresses the immune response by directly interacting with TLR9 and/or its ligands derived from environmental bacteria, thereby inhibiting TLR9-mediated stimulation. LF interacts with TLR2 and interferes with EBV-triggered TLR2-NF-*κ*B activation [15]. As TLR2 also recognizes the cell-wall components of Gram-positive bacteria [30], LF may suppress inflammation caused by the cell walls of Gram-positive bacteria. Similarly, LF binds to lipopolysaccharide (LPS), a cell-wall component of Gram-negative bacteria, and blocks the inflammatory response induced by the LPS–TLR4 interaction [31]. Therefore, LF may exert broad anti-inflammatory effects against bacteria.

pDCs lead the early phase of the antiviral immune response because they produce substantial amounts of IFN-α upon TLR7 stimulation and activate various immune cells, such as mDCs, T cells, B cells, and NK cells, and also display major histocompatibility complex (MHC) class II molecules, such as HLA-DR, along with co-stimulatory molecules, such as CD86, enabling them to present viral antigens to naïve T cells [32,33,34]. In a previous trial, oral ingestion of LF alleviated cold-like symptoms, and the activation of pDCs in the peripheral blood was proposed as a possible mechanism [11]. Orally ingested LF may be absorbed through the sublingual or lymphatic pathways [35,36] and subsequently influences immune cells in the peripheral blood; however, the concentration of absorbed LF cannot be high. Although oral administration of LF in mice enhances IFN-α/β production and activates T-helper cells, T-killer cells, and B cells in the Peyer’s patches in the small intestine [17,18], lymphocytes activated in GALT are primarily programmed to home to the small and large intestines; therefore, their effects on the upper respiratory tract may be limited [9]. In contrast, orally ingested LF can reach immune cells in the upper digestive tract, especially in the palatine tonsils, at high concentrations without dilution or digestion by digestive fluids. Immune cells in the TMCs activated by LF in this study were mainly pDCs, T-killer cells, and B cells. Immune responses in the palatine tonsils influence systemic immunity [1,9]; therefore, IFN-α produced by pDCs in the palatine tonsils may subsequently activate pDCs and other immune cells in the peripheral blood near the palatine tonsils. IgA-producing B cells may also home to mucosal tissues in the vicinity of the palatine tonsils, as lymphocytes activated in NALT preferentially home to the upper airway, where they contribute to the production of secretory IgA [9]. The distance from the palatine tonsils to the upper respiratory tract is shorter than that from the small intestine, a major gut immunity site; thus, the activation of pDCs and B cells in the palatine tonsils may affect the suppression of cold-like symptoms. This effect may be limited to orally ingested LF and is not expected for enteric-coated LF that passes through the palatine tonsils.

RT is characterized by repeated episodes of inflammation caused by bacterial or viral infections [5]. Despite the activation of pDCs, T-killer cells, and B cells, LF did not increase inflammatory cytokines IL-6 and TNF-α or Gd-IgA1, a risk factor of IgAN, under TLR7 stimulation. Additionally, LF decreased the activation of pDCs, mDCs, T-killer cells, T-helper cells, B cells, NK cells, inflammatory cytokines, and Gd-IgA1 under TLR9 stimulation. Therefore, LF may activate antiviral immunity without worsening inflammation during RT. In acute pharyngotonsillitis patients, 59% of *S. pyogenes* on the tonsillar surfaces was coated with human LF [37]. Furthermore, LF exhibits antimicrobial activity against *S. pyogenes* and inhibits its internalization into epithelial cells; gargling with LF solution decreases *S. pyogenes* in the tonsils [38,39]. As *S. pyogenes* is the major causative pathogen of RT, oral ingestion of LF may be useful for the treatment of RT due to its antimicrobial activity, as reported above, and its anti-inflammatory effects against CpG-DNA of *S. pyogenes*.

Glycosylation plays a critical role in the functional regulation of IgA. In IgAN, Gd-IgA1 is excessively produced in the tonsils and subsequently forms nephritogenic immune complexes with anti-glycan autoantibodies, which are deposited in glomerular mesangial cells, thereby inducing inflammation [6,40,41,42]. In addition, tonsillar expression of TLR7 and TLR9 is elevated in patients with IgAN and may contribute to the induction of inflammatory responses [43]. BAFF and APRIL, whose expression is induced downstream of TLR signaling, contribute to the overproduction of aberrantly glycosylated IgA (Gd-IgA1) [6]; thus, inhibition of TLR signaling and blockade of BAFF and APRIL are potential therapeutic strategies for IgAN to suppress the production of aberrantly glycosylated IgA and thereby reduce downstream inflammatory injury [44]. Despite the activation of pDCs and T-killer cells, LF did not increase inflammatory cytokine levels and Gd-IgA1 but decreased APRIL under TLR7 stimulation. Additionally, LF suppressed the activation of pDCs, mDCs, T-helper cells, T-killer cells, B cells, and NK cells; decreased IL-6, TNF-α and BAFF; and tended to decrease Gd-IgA1 and APRIL under TLR9 stimulation. Therefore, the oral ingestion of LF may help suppress the production of Gd-IgA1 and reduce inflammation in patients with IgAN.

Not only in IgAN but also in severe cases of COVID-19 and influenza, alterations in the glycosylation of plasma IgA have been reported, which may contribute to aberrant immunothrombosis and therefore to thromboembolic complications [45]. The palatine tonsils are one of the sites of respiratory viral infection where viruses persist for extended periods regardless of symptom status [46]. These observations raise the possibility that sustained immune stimulation within the tonsillar tissue due to viral infection may contribute to alterations in IgA glycosylation. Although LF did not alter the production of Gd-IgA1 from TMCs under TLR7 stimulation, the potential effects of LF on IgA glycosylation under such viral stimulation remain of interest.

In the present study, IgAN-derived TMCs showed higher pDC frequencies; IgA expression in B cells; and concentrations of BAFF, APRIL, and Gd-IgA1 in the culture supernatants than RT-derived TMCs. Similarly, compared with the TMCs of patients without IgAN, those of patients with IgAN show higher APRIL [47], BAFF, and IgA production under TLR9 stimulation [48]. Furthermore, the percentage of pDCs was significantly higher in the palatine tonsils of patients with IgAN than in those of patients with RT [49]. Our findings are consistent with those reported in previous studies and suggest that pDCs and B cells are more activated in the palatine tonsils of patients with IgAN than in those of patients with RT. Moreover, enhancement of the TLR7 response by LF was limited in TMCs derived from patients with IgAN compared with those from patients with RT in the present study. The TMCs of patients with IgAN exhibit higher TLR7 expression levels [43], and immune tolerance in the palatine tonsils is disrupted [6]; thus, TLR7 sensitivity is likely elevated. Moreover, plasma IFN-α levels are increased in patients with IgAN [50]. Accordingly, in IgAN-derived TMCs, the room for LF to further augment TLR7 sensitivity, enhance IFN-α production, and consequently amplify the activity of other immune cells may be limited.

This study has several limitations. First, donors of palatine tonsils were not healthy people but patients with RT and IgAN, and the number of tonsillectomy cases was limited, resulting in a relatively small sample size in this study. Given the substantial inter-individual variability in human immune function [51] and the possibility that immune responses differ across diseases, caution is warranted when extrapolating our findings to healthy populations or to patients with other conditions. Therefore, a larger number of samples will be necessary to more accurately evaluate the immunomodulatory effects of LF. Second, this study did not account for the age or sex of palatine tonsil donors. The composition and function of tonsillar immune cells are known to change with aging and also differ between sexes [52,53,54]. Although we compared TMCs derived from RT and IgAN patients, future studies should ensure greater alignment in donor age and sex to allow for a more precise interpretation of differences between the groups. In addition, this study was conducted in an exploratory manner, with the aim of comprehensively assessing the effects of LF on immune cells isolated from human palatine tonsils, and the analytical results may involve multiplicity. Consequently, re-evaluation focused on the specific parameter may be required to enhance the reliability of the results. Finally, this study did not examine the interactions among immune-cell populations. We speculated that IFN-α produced by pDCs mainly influences other immune cells in TMCs; however, to verify this hypothesis, it will be necessary to investigate whether depletion of pDCs from TMCs or neutralization of IFN-α attenuates the immunomodulatory effects of LF.

Immune cells in the palatine tonsils provide protection against pathogens while simultaneously maintaining tolerance to food antigens and commensal microorganisms [55]. According to our current data, LF tends to show immunostimulatory effects in TMCs without excessive inflammation without TLR stimulation and with TLR7 stimulation, whereas it shows immunosuppressive effects with TLR9 stimulation. Therefore, oral ingestion of LF may appropriately enhance the immune response against environmental ssRNA viruses while inducing immune tolerance against commensal bacteria in the palatine tonsils. This may also be the role of LF in tears, nasal secretions, saliva, and vaginal secretions on the mucosal surfaces of the eye, nasal cavity, oral cavity, and vagina. LF is also a constituent of bovine milk, which contains multiple immunity-modulating factors beyond LF, including immunoglobulins, milk oligosaccharides, and milk fat globule membrane (MFGM) components [56,57,58]. These milk-derived bioactives are considered to act as an integrated immunological system rather than as isolated factors, collectively shaping mucosal immune responses and maintaining immune homeostasis. Accordingly, LF may function in a complementary and coordinated manner with other milk-derived immune factors to regulate immune responses, and the immunomodulatory effects observed in the present study may reflect the physiological role of LF as one component of this integrated immunological system. Previous studies on food immunology have mainly focused on GALT but not on NALT. However, orally ingested LF may modulate immune responses not only in the small intestine but also in the upstream palatine tonsils. To the best of our knowledge, this is the first study to demonstrate the immunomodulatory effects of food ingredients on immune cells in the throat (palatine tonsils). Further in vivo and clinical studies are required to validate its efficacy, but our findings suggest the possibility that orally ingested LF modulates immune responses against environmental ssRNA viruses and bacteria in TMCs by regulating the TLR7 and TLR9 responses and balances cold-like symptoms, recurrent tonsillitis, and IgAN to a healthy condition.

## 4. Materials and Methods

### 4.1. Patients and Samples

Eligible donors of palatine tonsils were patients with recurrent tonsillitis (RT) or IgA nephropathy (IgAN) aged 18 years or older, excluding those receiving steroids or immunosuppressive agents and those deemed unsuitable by the principal investigator (Table 1). Sixteen patients who underwent tonsillectomy at the Asahikawa Medical University were enrolled in this study. Nephrologists diagnosed IgAN based on renal biopsy findings, including predominant IgA deposition in the mesangial area and proliferation of the mesangial cells and matrix. RT was identified in patients who experienced recurrent episodes of acute tonsillitis (three or more times per year), and all patients required tonsillectomy. All patients provided written informed consent for the therapy and tissue studies. The study protocol and informed consent documents were reviewed and approved by the Institutional Review Board of Asahikawa Medical University, Japan (Approval No. C24015).

### 4.2. Materials

The bovine LF used in the tonsillar mononuclear cell (TMC) culture experiment was purified from skimmed milk (97.2% purity; Morinaga Milk Industry, Tokyo, Japan) and contained 17.8 mg of iron per 100 g. FITC-labeled LF was generated by dissolving 1000 mg of bovine LF and 10 mg of FITC-I (Dojindo Laboratories, Kumamoto, Japan) in 0.1 M phosphate buffer (pH 8.0). The mixture was gently agitated for 2.5 h at room temperature in the dark, and the insoluble material was removed using centrifugation at 10,000× *g* for 5 min. The supernatant was subsequently dialyzed against phosphate buffer using a Spectra/Por 7 membrane (molecular weight cut-off = 10,000; Funakoshi, Tokyo, Japan) to eliminate the unreacted FITC and buffer components. The FITC-labeled LF was collected by freezing at −80 °C, followed by lyophilization. R-848, a TLR7/8 agonist, and class A CpG-ODN (ODN 2216), a TLR9 agonist, were purchased from InvivoGen (San Diego, CA, USA). All reagents were stored at −20 °C until use. LF, FITC-labeled LF, R-848, and CpG-ODN were aseptically dissolved in phosphate-buffered saline (PBS; Fujifilm Wako, Tokyo, Japan) and filtered through a 0.22 µm sterile filter membrane (Millipore, Burlington, MA, USA) before use in cell assays.

### 4.3. TMC Preparation

TMCs were prepared as described previously [47,48]. Tonsillar tissues obtained by tonsillectomy were washed with saline, mechanically fragmented into small pieces in saline, and filtered through gauze. TMCs were collected using gradient centrifugation with a Lymphoprep (Serumwerk Bernburg AG, Bernburg (Saale), Germany). The resulting cell fractions were washed five times with PBS, counted, and used for experiments on the same day. The viability of the cell suspension exceeded 90%.

### 4.4. TMC Culture

TMCs were seeded at a density of 1.25 × 10^6^ cells/mL in culture plates using Roswell Park Memorial Institute 1640 medium (Gibco, Grand Island, NY, USA) supplemented with 10% fetal bovine serum (Gibco), 100 U/mL penicillin, and 100 μg/mL streptomycin (Fujifilm Wako). TMCs were cultured overnight with 10 µg/mL R-848 or 1 µM CpG-ODN in the presence or absence of 100 µg/mL LF at 37 °C in a 5% CO_2_ incubator. The employed LF concentration was determined based on our previous study [13]. Following incubation, TMC culture supernatants were subjected to an enzyme-linked immunosorbent assay (ELISA), and TMCs were analyzed using flow cytometry. For TMCs intended for intracellular marker analysis using flow cytometry, brefeldin A (BD Biosciences, Franklin Lakes, NJ, USA), a protein transport inhibitor, was added after 5 h of culture to accumulate the cytokines produced in the cells. To evaluate the uptake of LF in TMCs, TMCs were cultured overnight with 100 µg/mL FITC-labeled LF and analyzed using flow cytometry. In addition, to examine the involvement of nucleolin, a LF receptor expressed on immune-cell populations, TMCs were pretreated with 5 µg/mL of a neutralizing antibody against nucleolin (Santa Cruz Biotechnology, Dallas, TX, USA) for 1h prior to the addition of FITC-labeled LF, as described in a previous study [13].

### 4.5. ELISA

Commercialized ELISA kits were used to measure IL-6 (Fujifilm Wako), TNF-α (Fujifilm Wako), Gd-IgA1 (Immuno-Biological Laboratories, Gunma, Japan), BAFF (Proteintech, Rosemont, IL, USA), and APRIL (Adipogen Life Sciences, Liestal, Switzerland) concentrations in TMC culture supernatants according to the manufacturers’ protocols.

### 4.6. Flow Cytometry

Following overnight incubation, as described in Section 4.4, the TMCs were washed with PBS and labeled with Horizon Fixable Viability Stain 780 (BD Biosciences) for 15 min at room temperature in the dark to exclude dead cells. After an additional wash with Stain Buffer (BD Biosciences), the Human BD Fc Block (BD Biosciences) was applied for 10 min on ice to prevent nonspecific antibody binding. The cells were then incubated for 30 min on ice and protected from light with a panel of fluorochrome-conjugated antibodies comprising CD1c-RB780, CD3-PreCP-Cy5.5, CD4-FITC, CD4-BV421, CD8-PE-Cy7, CD11c-BB515, CD11c-BV421, CD16-FITC, CD19-PE-Cy7, CD56-APC, CD56-BV421, CD69-PE, CD86-APC, CD123-PE-Cy7, CD183-APC, CD304-BB515, CD304-BB700 and HLA-DR-PE or their isotype controls (BD Biosciences). After surface staining, the cells were washed again with Stain Buffer and fixed with 4% paraformaldehyde (Muto Pure Chemical, Tokyo, Japan) for 20 min on ice in the dark. Fixed cells were washed with Stain Buffer and analyzed using a CytoFLEX instrument (Beckman Coulter, Brea, CA, USA). The instrument’s performance was standardized prior to acquisition using QC beads (Beckman Coulter), following the manufacturer’s recommendations. For intracellular marker detection (IFN-α, IFN-γ, BAFF, APRIL, and IgA), fixed cells were permeabilized with Perm/Wash Buffer (BD Biosciences) for 15 min on ice in the dark. The permeabilized cells were stained with IFN-α-Alexa Fluor 647, IFN-γ-PE, BAFF-PE (BD Biosciences), APRIL-APC, and IgA-APC (Miltenyi Biotec, Bergisch Gladbach, Germany) for 1 h in the dark. After staining, the samples were washed with Perm/Wash Buffer and analyzed using a CytoFLEX instrument. Flow cytometry data were processed using CytExpert 2.4 software (Beckman Coulter). In the flow cytometry analysis, dead cells were removed. and singlet mononuclear cells were gated from the total TMCs based on a forward scatter/side scatter plot. Lymphocyte subsets were defined as follows: pDCs = CD123+CD304+; mDCs = CD1c+CD11c+; T killer cells = CD3+CD8+; T helper cells = CD3+CD4; B cells = CD19+; NK cells = CD3−CD56+ (Figure S6). Immune-cell subset proportions in TMCs are expressed as the number of each subset per singlet mononuclear cell (%). The expression levels of activation markers in immune cells are reported as geometric mean fluorescence intensity (MFI). The uptake of FITC-labeled LF to TMCs was evaluated using the FITC MFI within each immune-cell subset. Fluorescence compensation was applied to correct for spectral overlap among the channels.

### 4.7. Statistical Analysis

All values are presented as mean and SD. Differences between two groups were analyzed using paired Student’s *t*-tests. Statistical significance was set at *p* < 0.05.

## 5. Conclusions

This study demonstrated that bovine LF modulates immune responses in tonsillar immune cells; it enhances immune activation under unstimulated conditions and TLR7 stimulation while suppressing immune activation under TLR9 stimulation.

## Figures and Tables

**Table 1:** Characteristics of TMC donors.

	RT	IgAN
Subjects, *n*	8	8
Men, *n*	4	2
Women, *n*	4	6
Age (SD) [range]	31.5 (10.5) [21–56]	38.4 (13.8) [26–68]

IgAN, immunoglobulin A nephropathy; RT, recurrent tonsillitis; TMCs, tonsillar mononuclear cells.

## Data Availability

Data underlying the results of this study can be obtained from the corresponding author upon request.

## Supplementary Materials

The following supporting information can be downloaded at: https://www.mdpi.com/article/10.3390/ijms27052442/s1.

## Abbreviations

The following abbreviations are used in this manuscript:

APRIL	A proliferation-inducing ligand
BAFF	B-cell activation factor
CpG-ODN	Geoxycytidyl-deoxyguanosine oligodeoxynucleotide
ELISA	Enzyme-Linked Immunosorbent Assay
GALT	Gut-associated lymphoid tissue
Gd-IgA1	Galactose-deficient IgA1
HLA-DR	Human leukocyte antigen–DR
IFN	Interferon
IgA	Immunoglobulin A
IgAN	Immunoglobulin A nephropathy
IL	Interleukin
LF	Lactoferrin
MALT	Mucosa-associated lymphoid tissue
mDCs	Myeloid dendritic cells
MFI	Geometric mean fluorescence intensity
NALT	Nasal-associated lymphoid tissue
NK	Natural killer
PBS	Phosphate-buffered saline
PBMCs	Peripheral blood mononuclear cells
pDCs	Plasmacytoid dendritic cells
RT	Recurrent tonsillitis
ssRNA	Single-stranded RNA
TLR	Toll-like receptor
TMCs	Tonsillar mononuclear cells
