# Supplementary material for: Bovine Lactoferrin Modulates Mononuclear Cell Activity in Human Palatine Tonsils
Source: Int J Mol Sci. 2026 Mar 6;27(5):2442. doi: 10.3390/ijms27052442 (PMC12985629; doi:10.3390/ijms27052442)
Supplement: Supplementary file 1 [file ijms-27-02442-s001.zip › ijms-4150196-supplementary.pdf]

Bovine lactoferrin modulates mononuclear cell activity in human palatine tonsils

Supplementary Material

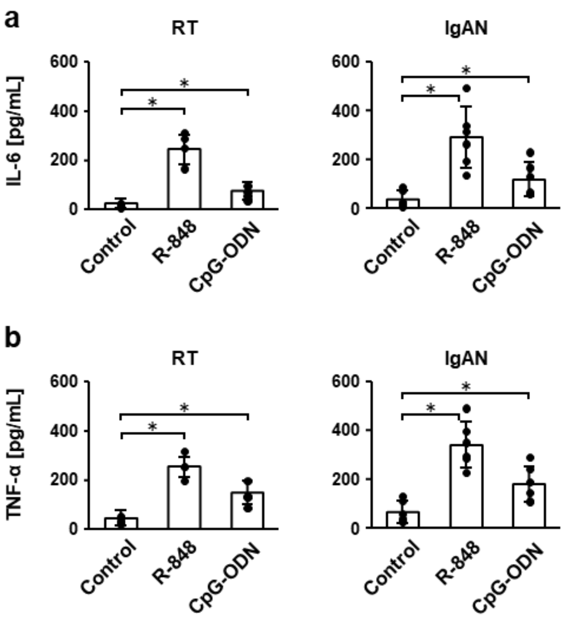

Figure S1. Inflammatory cytokine concentrations in the culture supernatants of TMCs. **(a)** IL-6 and **(b)** TNF-α concentrations under TLR7and TLR9 stimulation. After overnight incubation of TMCs from patients with RT and IgAN in the presence of 10 μg/mL R-848 or 1 μM CpG-ODN, IL-6 and TNF-α concentrations in culture supernatants were measured using ELISA. Values are presented as the mean and SD. Black circles represent individual values. n = 5–6. * Significantly different from the control group (p < 0.05 after Bonferroni correction following paired Student's *t*-test.). CpG-ODN, deoxycytidyl-deoxyguanosine oligonucleotide; IgAN, immunoglobullin A nephropathy; IL-6, Interleukin-6; TMCs, tonsillar mononuclear cells; TNF-α, tumor necrosis factor; RT, recurrent tonsillitis.

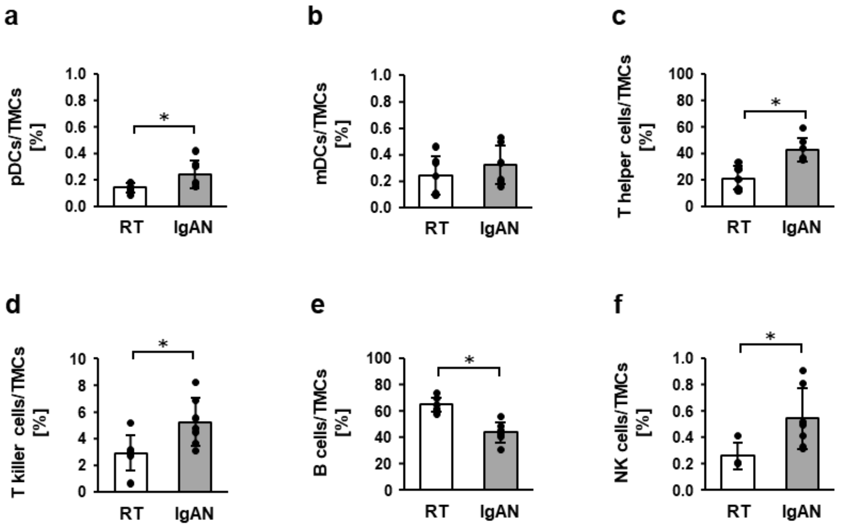

Figure S2. The cell composition of TMCs from patients with RT and IgAN. The frequency of **(a)** pDCs identified as $CD123^{+}CD304^{+}$ cells, **(b)** mDCs identified as CD1c+CD11c+ cells, **(c)** T-helper cells identified as CD3+CD4+ cells, **(d)** T-killer cells identified as CD3+CD8+ cells, **(e)** B cells identified as CD19+ cells, and **(f)** NK cells identified as $CD3^{-}CD56^{+}$ cells in TMCs. After overnight incubation of TMCs from RT and IgAN patients, the percentage of each immune cell in TMCs wasmeasured using flow cytometry. White bars represent the TMCs derived from the RT group, and gray bars represent the TMCs derived from the IgAN group. Values are presented as the mean and SD. Black circles represent individual values. n = 4–7. * Significantly different between the groups ($p < 0.05$, unpaired Student's *t*-test). IgAN, immunoglobullin A nephropathy; mDCs, myeloid dendritic cells; NK, natural killer; pDCs, plasmacytoid dendritic cells; RT, recurrent tonsillitis; TMCs, tonsillar mononuclear cells.

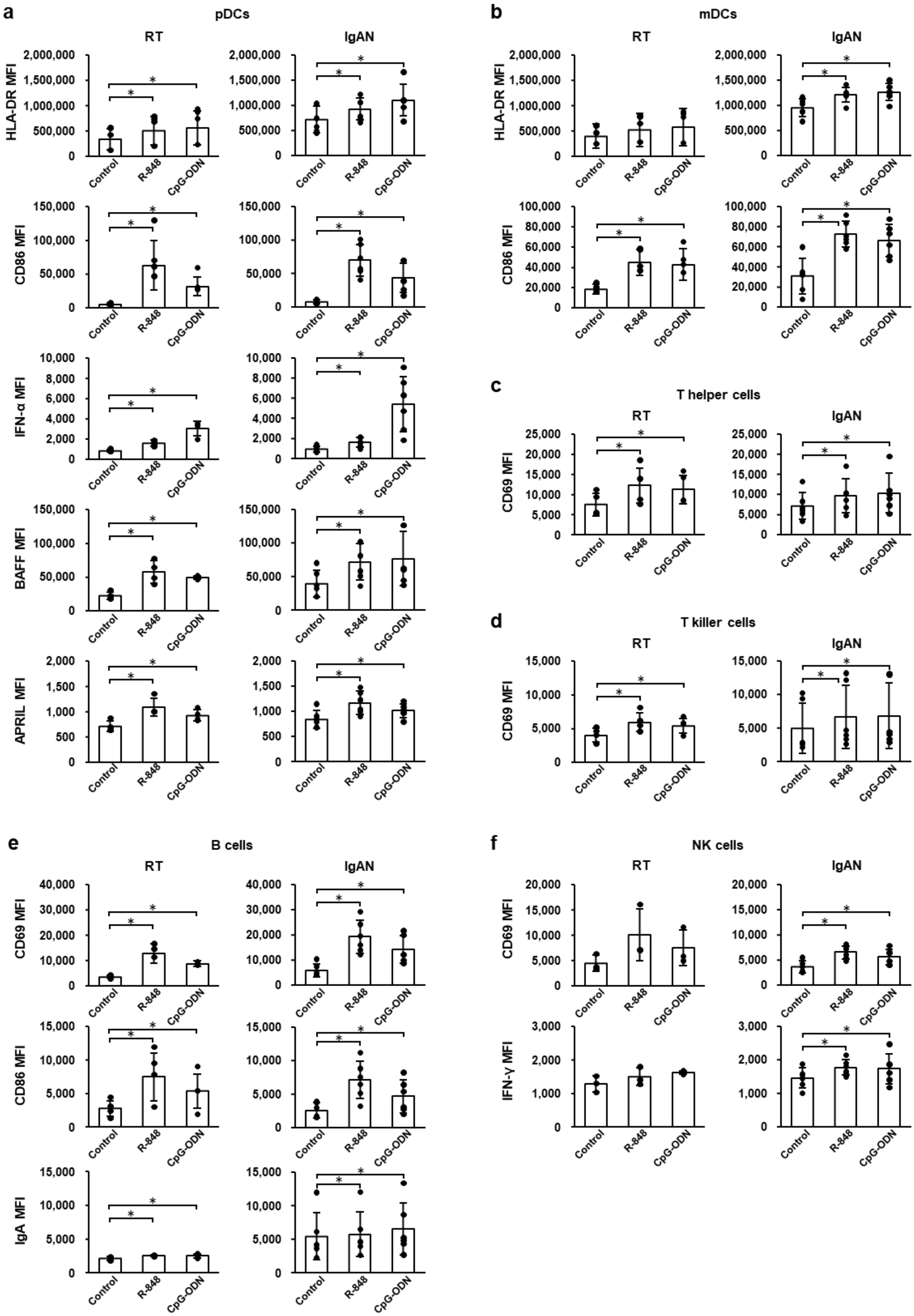
a
pDCs
RT
IgAN
HLA-DR MFI
CD86 MFI
IFN-α MFI
BAFF MFI
APRIL MFI
b
mDCs
RT
IgAN
HLA-DR MFI
CD86 MFI
c
T helper cells
RT
IgAN
CD69 MFI
d
T killer cells
RT
IgAN
CD69 MFI
e
B cells
RT
IgAN
CD69 MFI
CD86 MFI
IgA MFI
f
NK cells
RT
IgAN
CD69 MFI
IFN-γ MFI
Control
R-848
CpG-ODN
*

Figure S3. Expression levels of activation markers in the immune cells under TLR stimulation. **(a)** Cell-surface HLA-DR, CD86, intracellular IFN-α, BAFF and APRIL in pDCs. **(b)** Cell-surface HLA-DR and CD86 in mDCs. **(c)** Cell-surface CD69 in T-helper cells. **(d)** Cell-surface CD69 in T-killer cells. **(e)** Cell-surface CD69, CD86 and intracellular IgA in B cells. **(f)** Cell-surface CD69 and intracellular IFN-γ in NK cells. After overnight incubation of TMCs from RT and IgAN patients with 10 μg/mL R-848 or 1 μM CpG-ODN, the expression levels of immune cell activity markers were measured using flow cytometry. Values are presented as the mean and SD. Black circles represent individual values. n = 3–7. * Significantly different from the control group ($p < 0.05$ after Bonferroni correction following paired Student's *t*-test). CpG-ODN, deoxycytidyl-deoxyguanosine oligodeoxynucleotide; IgAN, immunoglobullin A nephropathy; mDC, myleoid dendritic cell; MFI, geometric mean fluorescence intensity; NK, natural killer; pDC, plasmacytoid dendritic cell; RT, recurrent tonsillitis; TMCs, tonsillar mononuclear cells.

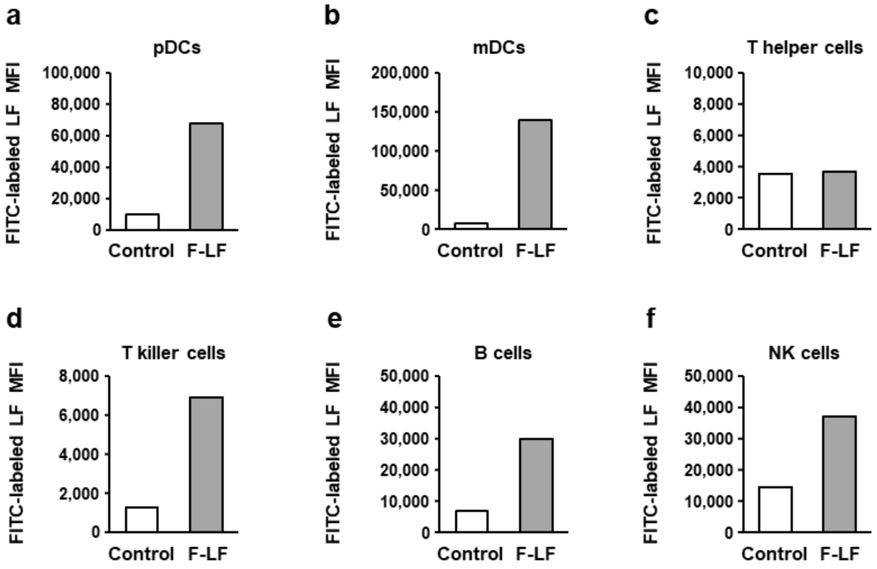

Figure S4. Incorporation of LF into TMCs. Fluorescein isothiocyanate (FITC) fluorescence signals of pDCs, mDCs, T-helper cells, T-killer cells, B cells and NK cells as measured using flow cytometry after overnight incubation of RT-derived TMCs with 100 μg/mL FITC-labeled LF. Values are presented as FITC MFI; n = 1. F-LF, FITC-labeled lactoferrin; mDC, myeloid dendritic cell; MFI, geometric mean fluorescence intensity; NK, natural killer; pDC, plasmacytoid dendritic cell; RT, recurrent tonsillitis; TMCs, tonsillar mononuclear cells.

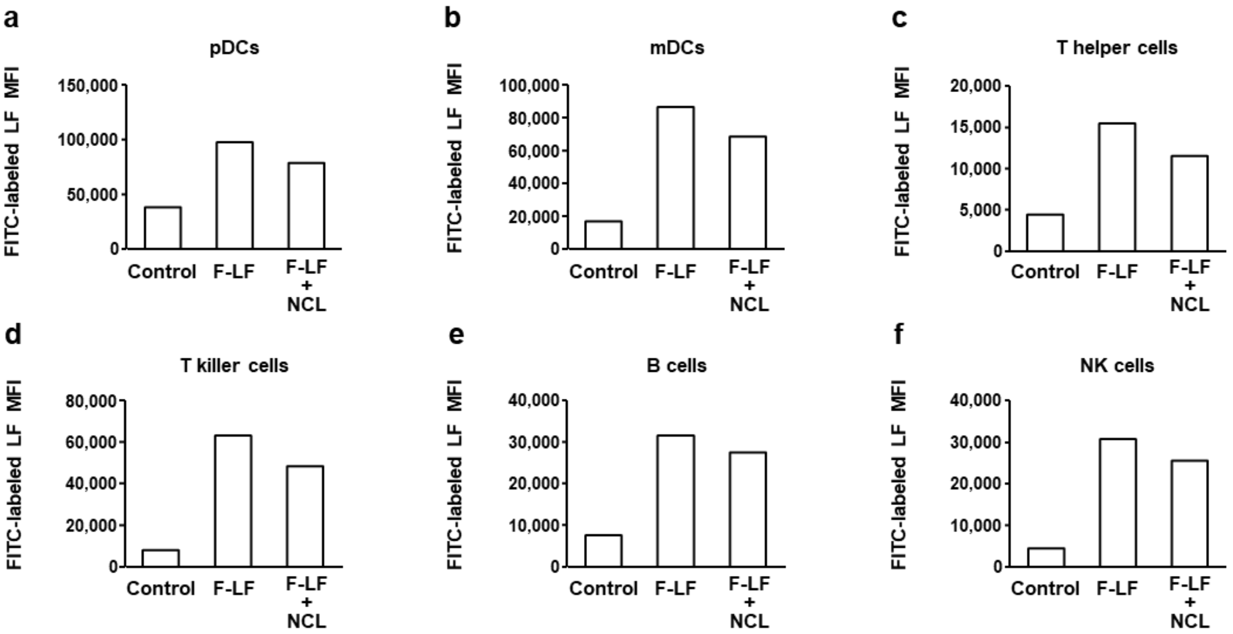

Figure S5. Effect of nucleolin neutralization on LF incorporation into TMCs. FITC fluorescence signals of pDCs, mDCs, T-helper cells, T-killer cells, B cells and NK cells as measured using flow cytometry after overnight incubation of IgAN-derived TMCs with 100 μg/mL FITC-labeled LF in the presence or absence of 5 μg/mL nucleolin-neutralizing antibody. Values are presented as FITC MFI; n = 1. F-LF, FITC-labeled lactoferrin; mDC, myeloid dendritic cell; MFI, geometric mean fluorescence intensity; NCL, nucleolin-neutralizing antibody; NK, natural killer; pDC, plasmacytoid dendritic cell; IgAN, immunoglobulin A nephropathy; TMCs, tonsillar mononuclear cells.

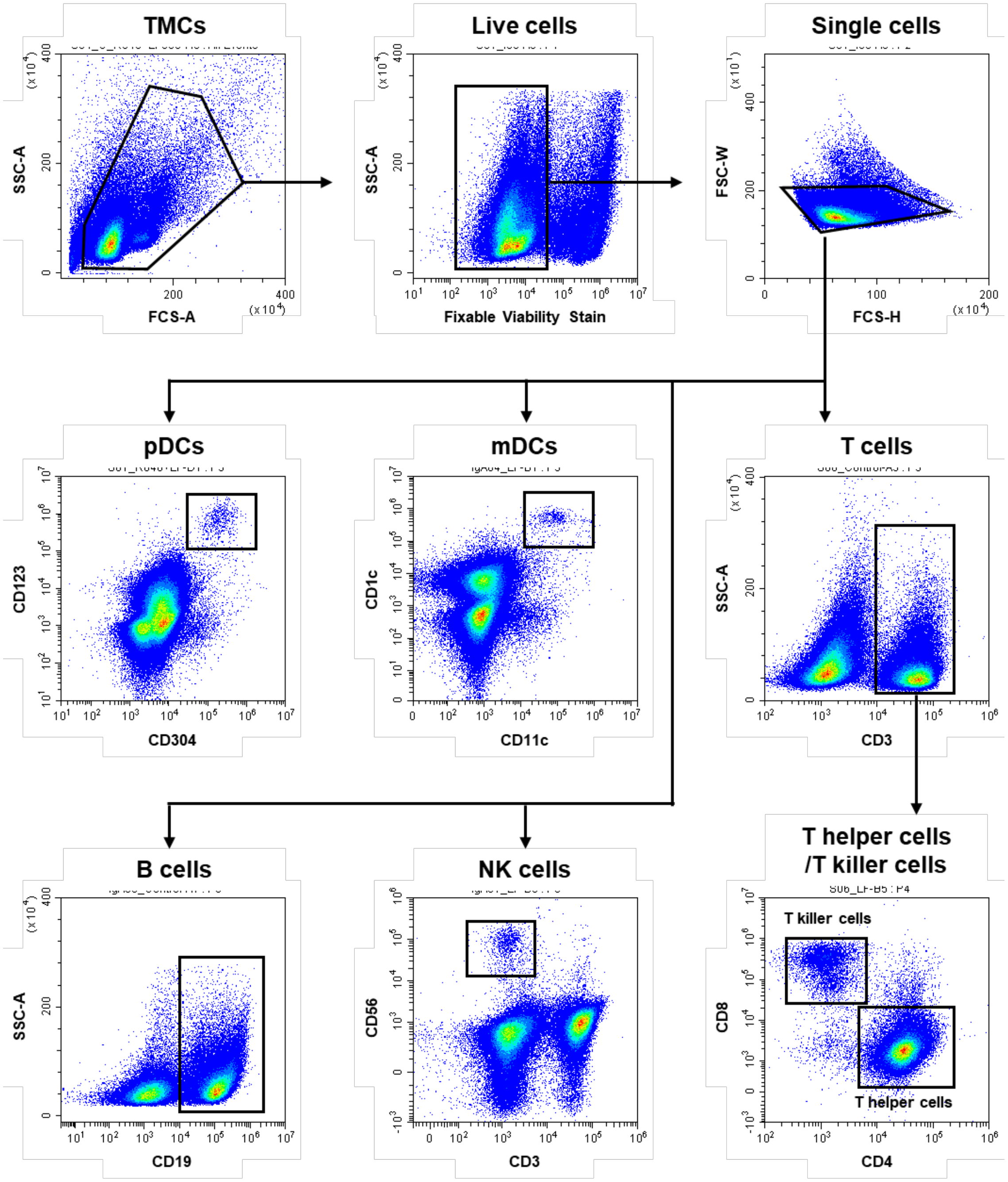

Figure S6. Gating strategy to observe pDCs, mDCs, T-helper cells, T-killer cells, B cells and NK cells in TMCs.
